# Differential motivational pathways to flow experience in competitive sailing

**DOI:** 10.3389/fpsyg.2026.1784085

**Published:** 2026-05-07

**Authors:** Cheng-Yu Hsu

**Affiliations:** Department of Recreational Sport, National Taiwan University of Sport, Taichung, Taiwan

**Keywords:** competitive sport, motivation, performance, psychology, sailing regatta

## Abstract

**Introduction:**

Understanding how motivational orientations shape flow experience is central to sport and performance psychology, particularly in competitive contexts characterized by high task demands and environmental uncertainty. Although motivation has long been regarded as a key antecedent of flow, limited empirical research has examined how different motivational orientations differentially influence flow experience in sailing regattas.

**Methods:**

This study investigated the relationships between participation motivations and flow experience among competitive sailing regatta participants. Flow experience was operationalized through three core experiential dimensions: concentration, perceived control, and time distortion. Data were collected from 192 sailors with recent regatta experience and analyzed using partial least squares structural equation modeling (PLS-SEM).

**Results:**

The results reveal that competition motivation and novelty–exploration motivation exert significant positive effects on all three dimensions of flow experience. In contrast, relaxation motivation demonstrates a consistent and significant negative association with concentration, perceived control, and time distortion. Challenge motivation does not show a significant direct effect on any flow dimension, suggesting that perceived challenge may be normalized within highly competitive sailing environments. Environmental experience and social interaction motivations exhibit selective positive effects on specific flow dimensions.

**Discussion:**

These findings highlight the context-dependent nature of motivational influences on flow experience in competitive sailing. While achievement-oriented and novelty-driven motivations facilitate deep immersion and optimal engagement, relaxation-oriented motives appear psychologically incompatible with the heightened attentional and control demands of competition. The study contributes to sport psychology by clarifying how distinct motivational orientations differentially shape flow experience in competitive maritime sport settings, offering practical implications for event design, participant segmentation, and performance-oriented sport management.

## 1 Introduction

Understanding how motivational orientations shape flow experience is a central concern in sport and performance psychology, particularly in competitive contexts characterized by high task demands and environmental uncertainty. Flow experience commonly defined by deep concentration, a strong sense of control, and altered time perception, represents an optimal psychological state that supports sustained engagement and performance under pressure (Csikszentmihalyi, 1990; Jackson and Marsh, 1996). Within sport settings, flow has been consistently associated with enhanced intrinsic motivation, performance quality, and continued participation, highlighting its importance for understanding athletes’ psychological experiences during competition (Jackson and Csikszentmihalyi, 1999; Swann et al., 2012).

Motivation has long been recognized as a key antecedent of flow experience. From a sport psychology perspective, motivational orientations reflect individuals’ underlying reasons for engagement and shape how attentional resources, cognitive control, and effort regulation are mobilized during task performance (Vallerand and Losier, 1999). Empirical research suggests that achievement-oriented motivations, such as competition and skill development, facilitate flow by encouraging individuals to actively invest effort and align perceived demands with personal capabilities. In contrast, motivations emphasizing relaxation or recovery may be psychologically incongruent with the heightened attentional and control demands required for flow in competitive environments (Jackson et al., 2001; Swann et al., 2015). Importantly, flow theory emphasizes the balance between perceived challenge and skill rather than challenge alone, suggesting that the role of challenge motivation in competitive contexts may be more complex than traditionally assumed (Csikszentmihalyi, 1990).

Despite substantial theoretical attention, empirical findings regarding the motivational antecedents of flow remain mixed, particularly in highly competitive sport settings. Recent sport psychology studies indicate that when performance demands are consistently high, perceived challenge may become normalized or even interpreted as performance pressure, thereby weakening its direct relationship with flow experience (Peifer et al., 2014; Swann et al., 2015). This highlights the need to examine how different participation motivations exert differential effects on specific components of flow experience within demanding sport contexts, rather than assuming uniform positive effects across motivational dimensions.

Sailing events are increasingly recognized as an important component of the emerging blue economy and maritime tourism development. As coastal regions seek to diversify tourism and recreation opportunities, sailing regattas and related maritime sport events have been promoted as mechanisms for stimulating local tourism, enhancing destination attractiveness, and fostering sustainable ocean-based recreation industries (Jaimangal-Jones et al., 2023; Jones and Navarro, 2018; Fazil et al., 2025). Within this context, understanding the psychological experiences of participants in sailing competitions is not only relevant to sport psychology but also contributes to broader discussions regarding the role of maritime sport events in supporting the development of the blue economy.

Competitive sailing regattas provide a suitable context for such examination. Sailing competitions involve sustained cognitive demands, environmental uncertainty, and continuous real-time decision-making, requiring participants to regulate attention, maintain control over vessel handling, and adapt to rapidly changing wind and course conditions. These characteristics make sailing regattas an appropriate performance-based setting for investigating the psychological mechanisms underlying flow experience in sport. Understanding sailors’ flow experience is particularly valuable, as intense concentration, perceived control, and altered time perception during competition have been linked to enhanced satisfaction, continued participation, and positive experiential outcomes (Armbrecht and Andersson, 2020; Li et al., 2025).

Prior research across outdoor and water-based recreation contexts consistently demonstrates that participation motivation is a multidimensional and context-dependent construct. Lee et al. (2007) identified relaxation, social interaction, challenge, competition, and novelty as key motivations among paddling participants, with variations across levels of recreation specialization. Similarly, O’Connell (2010) reported that sea kayaking motivations encompass achievement, excitement, and experiential exploration, while also varying by age, gender, and experience. Galloway (2012) further demonstrated that different water-based activities generate distinct motivational configurations, emphasizing the role of activity characteristics. Studies in related contexts, such as shelter camping and nature-based recreation, have likewise shown that motivations including nature contact, relaxation, skill development, and social interaction vary according to group composition and experience level (Kristensen et al., 2021; Whiting et al., 2017). Collectively, this body of research indicates that participation motivation in outdoor and water-based activities is inherently multidimensional and strongly shaped by activity demands and environmental conditions.

Based on this literature, adopting the motivational framework proposed by Lee et al. (2007) provides a theoretically grounded and contextually appropriate foundation for examining participation motivation in sailing regatta events. Their framework encompasses six intrinsic motivational dimensions: challenge, competition, novelty and exploration, natural environmental experience, social interaction, and relaxation, which closely align with the psychological drivers relevant to sailing competition. Importantly, distinguishing between challenge motivation (self-referenced striving for personal improvement) and competition motivation (performance comparison with others) allows for a more nuanced examination of their differential effects on flow experience in competitive sport contexts.

Flow experience in water-based and adventure recreation has commonly been operationalized using three core dimensions: concentration and absorption, sense of control, and altered perception of time. Empirical studies across scuba diving, white-water rafting, and surfing contexts have consistently demonstrated that these dimensions reliably capture optimal experiential states characterized by deep task immersion and psychological engagement (Wu and Liang, 2011; Cheng and Lu, 2015; Cheng et al., 2024). Although flow was originally conceptualized as a multidimensional construct, concentration, perceived control, and time distortion have emerged as the most frequently adopted and psychometrically robust dimensions across sport and recreation research.

Applying these three dimensions to competitive sailing is theoretically justified. Sailing competitions require sustained attention to environmental cues, continuous regulation of motor actions, and rapid tactical decision-making, while often inducing a distorted sense of time during races. These psychological characteristics closely correspond to the core processes underlying flow experience identified in prior sport psychology research. Accordingly, the present study examines how participation motivations influence sailors’ flow experience by adopting the six motivational dimensions proposed by Lee et al. (2007) as independent variables and the three core dimensions of flow experience: concentration, sense of control, and time distortion, as dependent variables. By doing so, this study aims to clarify the context-dependent psychological mechanisms linking motivation and flow experience in competitive sailing settings.

Building on this conceptualization, the present study examines how participation motivations influence sailors’ flow experience in competitive sailing contexts. Drawing on the motivational framework proposed by Lee et al. (2007), six participation motivations are considered: challenge, competition, novelty and exploration, environmental experience, social interaction, and relaxation. These motivational dimensions are treated as independent variables, while the three flow dimensions, concentration, sense of control, and time distortion, are treated as dependent variables.

Based on flow theory and prior sport psychology research, the study proposes that participation motivations may exert differential effects on these experiential components of flow. Therefore, a conceptual research model is developed to examine how different motivational orientations influence the three dimensions of flow experience during sailing regatta participation. By doing so, this study aims to clarify the context-dependent psychological mechanisms linking motivation and flow experience in competitive sailing settings.

Based on the conceptual framework, the present study examines how different participation motivations influence sailors’ flow experience in competitive sailing contexts. Participation motivations reflect individuals’ underlying reasons for engaging in sport activities and may shape how attentional resources, cognitive engagement, and behavioral efforts are directed during performance. Previous research suggests that different motivational orientations may facilitate or inhibit the emergence of flow experiences depending on how well they align with the psychological demands of the activity.

In competitive sailing environments, motivations related to challenge, competition, exploration, environmental engagement, and social interaction may stimulate cognitive involvement and attentional focus, thereby enhancing the likelihood of experiencing flow. Conversely, relaxation-oriented motivations may not align well with the high levels of concentration and performance demands required in competitive contexts. Therefore, the present study proposes that different motivational orientations may exert distinct influences on the three experiential dimensions of flow experience: concentration, sense of control, and time distortion.

**FIGURE 1:**
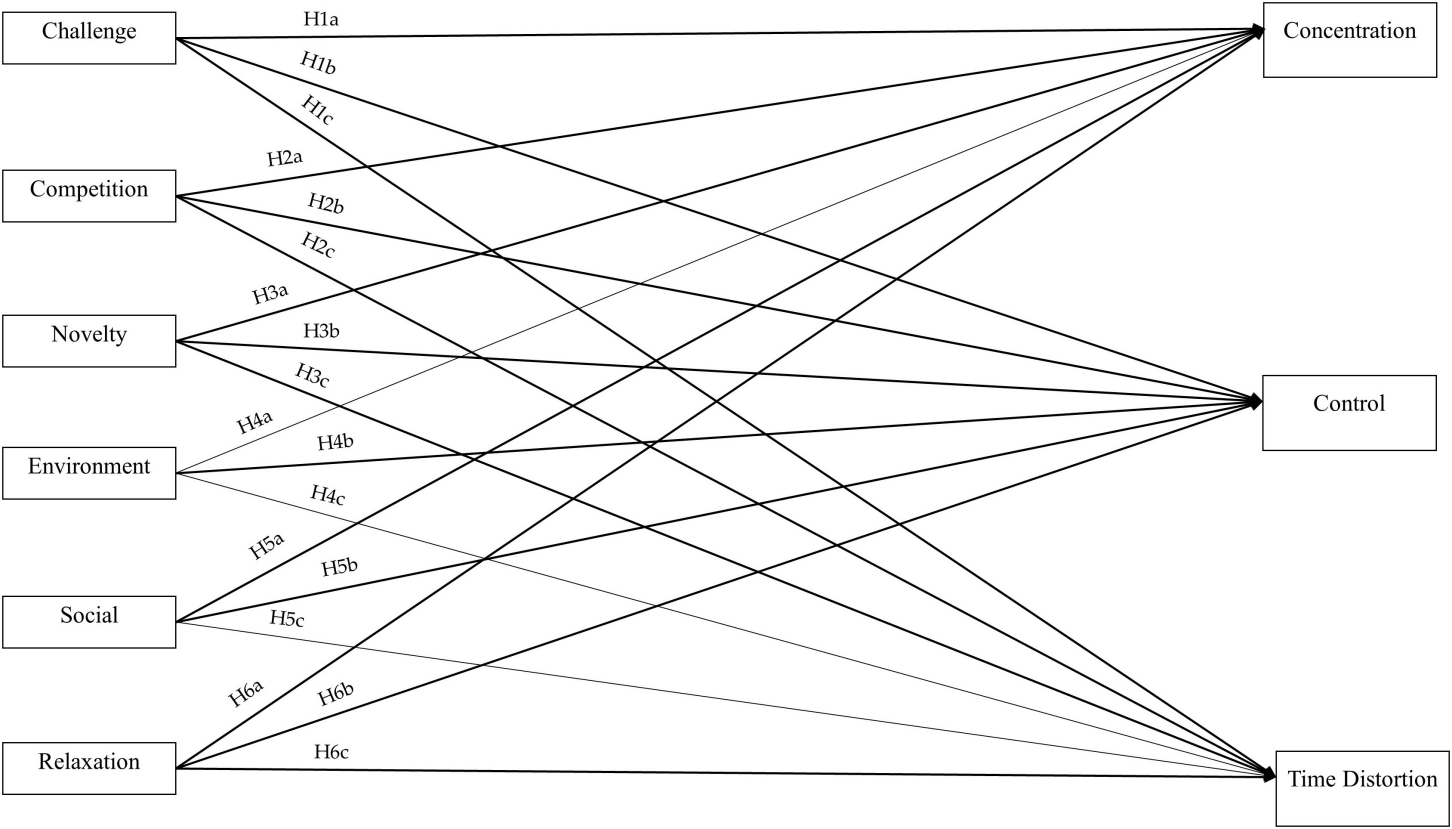
Proposed research model.

Based on this reasoning, the following hypotheses are proposed. The proposed research model is shown in Figure 1.

*H1a:* Challenge motivation significantly influences sailors’ concentration during sailing competitions.

*H1b:* Challenge motivation significantly influences sailors’ sense of control during sailing competitions.

*H1c:* Challenge motivation significantly influences sailors’ time distortion during sailing competitions.

*H2a:* Competition motivation significantly influences sailors’ concentration during sailing competitions.

*H2b:* Competition motivation significantly influences sailors’ sense of control during sailing competitions.

*H2c:* Competition motivation significantly influences sailors’ time distortion during sailing competitions.

*H3a:* Novelty and exploration motivation significantly influences sailors’ concentration during sailing competitions.

*H3b:* Novelty and exploration motivation significantly influences sailors’ sense of control during sailing competitions.

*H3c:* Novelty and exploration motivation significantly influences sailors’ time distortion during sailing competitions.

*H4a:* Environmental (nature) experience motivation significantly influences sailors’ concentration during sailing competitions.

*H4b:* Environmental (nature) experience motivation significantly influences sailors’ sense of control during sailing competitions.

*H4c:* Environmental (nature) experience motivation significantly influences sailors’ time distortion during sailing competitions.

*H5a:* Social interaction motivation significantly influences sailors’ concentration during sailing competitions.

*H5b:* Social interaction motivation significantly influences sailors’ sense of control during sailing competitions.

*H5c:* Social interaction motivation significantly influences sailors’ time distortion during sailing competitions.

*H6a:* Relaxation motivation significantly influences sailors’ concentration during sailing competitions.

*H6b:* Relaxation motivation significantly influences sailors’ sense of control during sailing competitions.

*H6c:* Relaxation motivation significantly influences sailors’ time distortion during sailing competitions.

## 2 Methods

### 2.1 Data collection

To empirically test the proposed research model, data were collected from participants involved in competitive sailing regattas in Taiwan, an emerging sailing context in Asia. Taiwan was selected as the research setting because it represents a developing sailing market with regularly organized regatta events, providing an appropriate context for examining participation motivations and flow experience in competitive sailing environments.

A purposive sampling strategy was employed to recruit individuals with direct sailing competition experience. Questionnaires were distributed through sailing clubs, sailing associations, and relevant community networks. Of the 250 questionnaires distributed, 192 valid responses were retained after excluding incomplete or invalid cases. All respondents had participated in at least one sailing regatta within the previous year, ensuring the relevance of the sample to the research objectives. The sample consisted predominantly of male participants (90.1%), reflecting the current demographic structure of competitive sailing participation in Taiwan. Most respondents were aged between 41 and 50 years (25.0%), and over half held an undergraduate degree (57.2%). Overall, the sampling and data collection procedures ensured that the respondents possessed appropriate experience and knowledge relevant to competitive sailing participation.

Prior to statistical analysis, the dataset was screened for missing values and data completeness. Questionnaires with incomplete responses were removed using listwise deletion during the data cleaning process. After this screening procedure, 192 valid responses were retained for the final analysis. The final dataset contained no missing values for the variables included in the PLS-SEM analysis; therefore, no data imputation procedures were required.

The adequacy of the sample size was evaluated based on established guidelines for Partial Least Squares Structural Equation Modeling (PLS-SEM). According to the commonly applied 10-times rule, the minimum sample size should be at least ten times the maximum number of structural paths directed toward any endogenous construct in the model (Hair et al., 2019). In the present study, six motivational constructs predict each dimension of flow experience, indicating a minimum sample size requirement of 60 observations. The final sample size of 192 therefore substantially exceeds this threshold and is considered sufficient for estimating the proposed structural model. Furthermore, previous methodological studies indicate that PLS-SEM is well suited for research involving relatively small to medium sample sizes and complex structural relationships (Hair et al., 2019; Sarstedt et al., 2017). In addition, the obtained sample size provides sufficient statistical power to detect medium effect sizes in structural equation modeling analyses (Cohen, 2013).

To minimize the potential influence of common method bias, several procedural remedies were implemented during the survey process. Participation was entirely voluntary, responses were collected anonymously, and respondents were assured that their answers would remain confidential and used solely for research purposes. In addition, the questionnaire emphasized that there were no right or wrong answers, thereby reducing evaluation apprehension and the likelihood of socially desirable responses.

### 2.2 Instrument

The measurement instrument used in this study was developed based on established frameworks of participation motivation and flow experience in outdoor and water-based recreation research, with contextual adaptations for sailing regatta participation. The participation motivation scale was primarily adapted from Lee et al. (2007) and further in-formed by related studies, including O’Connell (2010), Galloway (2012), Kristensen et al. (2021), and Whiting et al. (2017). Considering the unique characteristics of sailing competitions, such as environmental uncertainty, technical demands, and competitive interaction, the scale items were refined to reflect the sailing regatta context. The final participation motivation scale consisted of six dimensions, with three items measuring each dimension.

Flow experience was measured using a scale adapted from prior studies on water-based and adventure activities, including Cheng et al. (2024), Wu and Liang (2011), and Cheng and Lu (2015). In line with these studies, flow experience was conceptualized as a multidimensional construct encompassing control, concentration, and time distortion. The measurement items were modified to capture the experiential characteristics of sailing regatta participation. The final flow experience scale comprised three dimensions, with three items for each dimension. All items in the questionnaire were measured using a 5-point Likert scale, ranging from 1 = strongly disagree to 5 = strongly agree.

As the original measurement items were developed in English, a translation and back-translation procedure was employed to ensure linguistic equivalence and content validity. First, the items were translated into Chinese by a bilingual researcher with expertise in sport and recreation studies. A second independent bilingual researcher then back-translated the Chinese version into English. The original and back-translated English versions were subsequently compared, and any discrepancies were discussed and resolved through consensus among the researchers. This procedure helped ensure conceptual consistency and clarity of the measurement items in the sailing regatta context (Memon et al., 2017).

While this process ensured linguistic equivalence between the original and translated versions, the scale was not fully culturally adapted to the Taiwanese context. The primary objective of the translation procedure was to preserve the conceptual meaning of the original measurement framework rather than to develop a culturally specific instrument. The measurement items and constructs are shown in Table 1.

**TABLE 1:** The survey questionnaire items.

Construct	Item
Challenge	CHL1: *I participate in sailing regattas to experience personal challenge.*
	CHL2: *I participate in sailing regattas to improve my sailing skills.*
	CHL3: *I participate in sailing regattas to test my sailing-related abilities and skills.*
Competition	COM1: *I participate in sailing regattas to experience what it feels like to be a competitive sailor.*
	COM2: *I participate in sailing regattas to experience competition.*
	COM3: *I participate in sailing regattas to compete and exchange skills with other sailors.*
Novelty	NOV1: *I participate in sailing regattas to satisfy my curiosity.*
	NOV2: *I participate in sailing regattas to explore unfamiliar things.*
	NOV3: *I participate in sailing regattas to experience something new.*
Environment	ENV1: *I participate in sailing regattas to learn more about the natural environment.*
	ENV2: *I participate in sailing regattas to be close to nature.*
	ENV3: *I participate in sailing regattas to place myself in a natural environment.*
Social	SOC1: *I participate in sailing regattas to be with my friends.*
	SOC2: *I participate in sailing regattas to meet new friends.*
	SOC3: *I participate in sailing regattas to be with people who share similar interests.*
Relaxation	RLX1: *I participate in sailing regattas to get away from everyday concerns.*
	RLX2: *I participate in sailing regattas to release tension and anxiety.*
	RLX3: *I participate in sailing regattas to achieve mental relaxation.*
Concentration	CNT1: *I do not think about anything else when I am sailing.*
	CNT2: *I become highly concentrated when sailing.*
	CNT3: *I become completely absorbed in sailing.*
Sense of Control	CON1: *When I am sailing, I feel completely in control.*
	CON2: *I feel that everything is under control when I am sailing.*
	CON3: *I feel that the sailing equipment is fully under my control.*
Time distortion	TDS1: *Time seems to pass quickly when I am sailing.*
	TDS2: *Rapid changes during sailing make me feel that time passes quickly.*
	TDS3: *When I am sailing, my perception of time feels different from usual.*

### 2.3 Data analysis

In this study, Partial Least Squares Structural Equation Modeling (PLS-SEM) was conducted using SmartPLS 4.0 to evaluate both the measurement and structural models (Sarstedt and Cheah, 2019). Following established guidelines, a two-stage analytical procedure was adopted, beginning with an assessment of the measurement model, followed by hypothesis testing of the structural relationships (Hair, 2014). To evaluate internal consistency reliability, Cronbach’s alpha and Composite Reliability (CR) were examined, with values exceeding the recommended threshold of 0.70 indicating satisfactory reliability (Hair et al., 2019). Convergent validity was assessed through standardized factor loadings and Average Variance Extracted (AVE), with AVE values above 0.50 demonstrating adequate variance explained by each construct. Discriminant validity was evaluated using the Heterotrait–Monotrait Ratio (HTMT), which has been shown to be more sensitive than the Fornell–Larcker criterion. HTMT values below 0.90 were interpreted as evidence of sufficient discriminant validity. In addition, multicollinearity was assessed using the Variance Inflation Factor (VIF), with values below 5 indicating that collinearity did not pose a threat to the estimation of structural paths (Hair, 2014; Hair et al., 2019). After establishing the adequacy of the measurement model, the structural model was examined. Path coefficients (β) were used to assess the strength and direction of the hypothesized relationships between participation motivations and flow experience. Statistical significance was evaluated through a bootstrapping procedure with 5,000 resamples, using a *p*-value threshold of < 0.05. The model’s explanatory power was assessed using the coefficient of determination (*R*^2^), representing the proportion of variance explained in the endogenous constructs (Carrión et al., 2017; Hair, 2014; Sarstedt et al., 2017).

## 3 Results

### 3.1 Measurement model

To verify the psychometric properties of the scales, the measurement model was rigorously scrutinized through assessments of internal consistency, convergent validity, and discriminant validity. The primary metrics, encompassing standardized outer loadings, Composite Reliability (CR), Average Variance Extracted (AVE) in Table 2. The results demonstrate that all outer loadings surpassed the conservative threshold of 0.70, indicating a strong association between the latent constructs and their respective indicators. Furthermore, the AVE values for all constructs exceeded the 0.50 benchmark, confirming that the measurement model possesses robust convergent validity, as the constructs explain a majority of the variance in their indicators. Finally, discriminant validity was established using the Heterotrait–Monotrait Ratio (HTMT) criterion, which is considered a more stringent test than traditional methods recommended by Henseler et al. (2015). Following established guidelines, HTMT values below the threshold of 0.90 were considered indicative of adequate discriminant validity. As shown in Table 3, all HTMT values were below the recommended threshold, indicating satisfactory discriminant validity among the constructs.

**TABLE 2:** Assessment of reliability and convergent validity of construct.

Construct	Item	Loading	CR	Cronbach’s α	AVE
Challenge	CHL1	0.803	0.880	0.799	0.711
	CHL2	0.774			
	CHL3	0.943			
Competition	COM1	0.915	0.844	0.733	0.645
	COM2	0.740			
	COM3	0.743			
Novelty	NOV1	0.881	0.903	0.844	0.762
	NOV2	0.916			
	NOV3	0.819			
Environment	ENV1	0.795	0.906	0.850	0.756
	ENV2	0.901			
	ENV3	0.907			
Social	SOC1	0.771	0.860	0.843	0.673
	SOC2	0.789			
	SOC3	0.896			
Relaxation	RLX1	0.861	0.905	0.765	0.760
	RLX2	0.884			
	RLX3	0.870			
Concentration	CNT1	0.913	0.840	0.905	0.840
	CNT2	0.918			
	CNT3	0.918			
Control	CON1	0.886	0.853	0.786	0.853
	CON2	0.948			
	CON3	0.935			
Time distortion	TDS1	0.763	0.874	0.786	0.698
	TDS2	0.893			
	TDS3	0.846			

CR, Composite Reliability; Cronbach’s α, Cronbach’s alpha; AVE, Average Variance Extracted.

**TABLE 3:** Assessment of discriminant validity using HTMT.

Construct	CHL	COM	NOV	ENV	SOC	RLX	CNT	CON
Challenge (CHL)	0.643	0.331	0.695	0.511	0.305	0.325	0.691	0.804
Competition (COM)								
Novelty (NOV)	0.227							
Environment (ENV)	0.229	0.198						
Social (SOC)	0.183	0.101	0.647					
Relaxation (RLX)	0.247	0.238	0.231	0.333				
Concentration (CNT)	0.418	0.366	0.617	0.279	0.420			
Control (CON)	0.310	0.436	0.536	0.404	0.324	0.096		
Time distortion (TDS)	0.453	0.552	0.599	0.342	0.268	0.273	0.843	

### 3.2 Structural model and hypothesis test

#### 3.2.1 Common method bias and collinearity diagnostics

Common method bias was assessed using the full collinearity variance inflation factor (VIF) approach recommended by Hair et al. (2019) and Kock (2015). This method is suitable for PLS-SEM and allows researchers to diagnose the potential presence of common method bias in addition to multicollinearity. All full collinearity VIF values were below the recommended threshold of 5, indicating that common method bias is unlikely to pose a serious threat to the validity of the results. Furthermore, several procedural remedies were implemented during data collection, including voluntary participation, anonymous responses, confidentiality assurances, and instructions emphasizing that there were no right or wrong answers. These procedural safeguards helped reduce evaluation apprehension and the likelihood of socially desirable responding.

#### 3.2.2 Path analysis results

**FIGURE 2:**
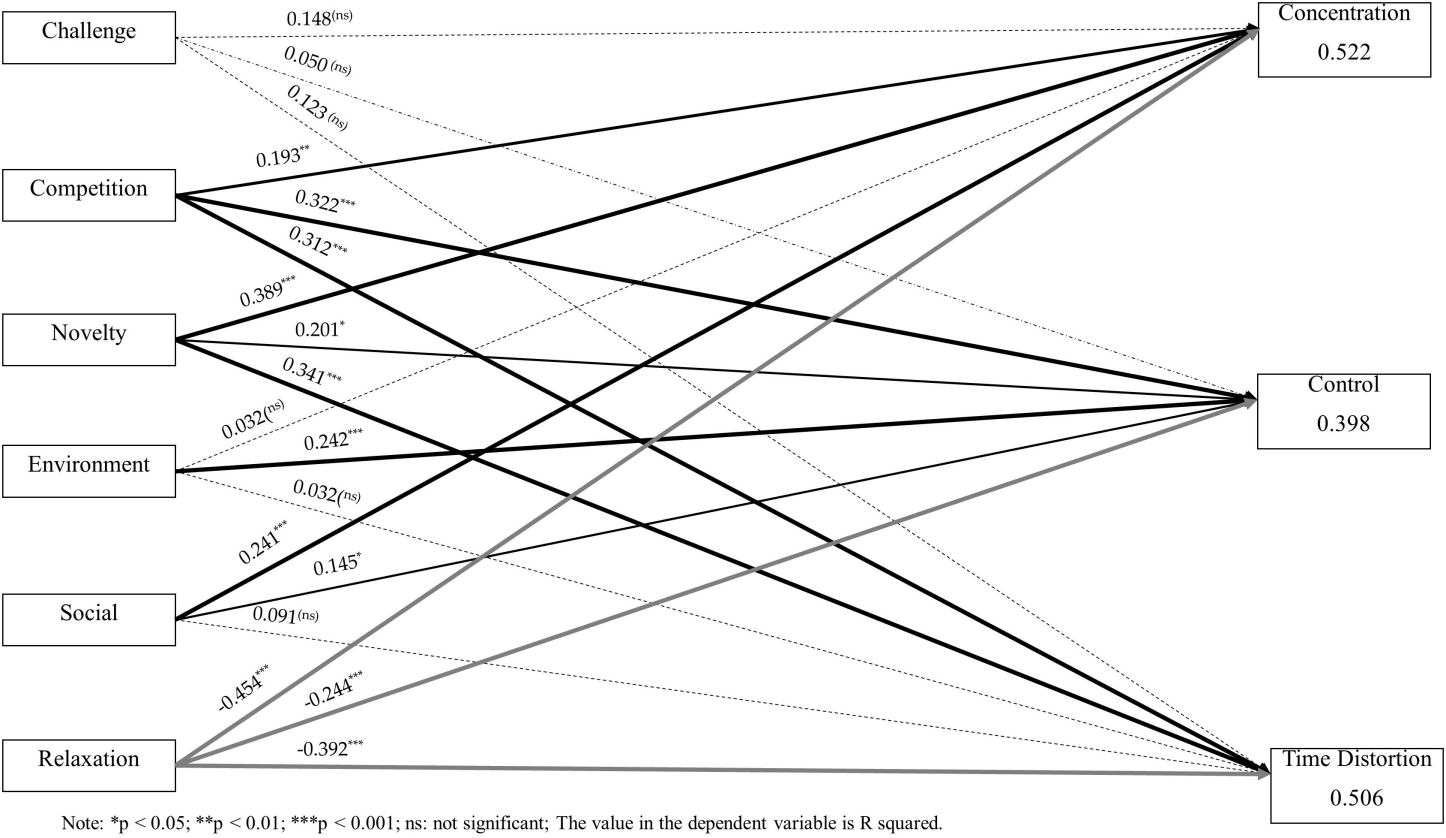
Structural model results. *p< 0.05; **p < 0.01; ***p < 0.001; ns, not significant. The value in the dependent variable is R squared.

The results of the PLS path analysis, including the standardized path coefficients (β) and their respective significance levels (*p*-value), are illustrated in Figure 2 and summarized in Table 4. The empirical findings for each hypothesized path are detailed as follows:

**TABLE 4:** Assessment of the structural model.

Hypothesis	Relationship	β	*p*-value
H1a	CHL CNT	0.148	0.064 (ns)
H1b	CHL CON	0.050	0.468 (ns)
H1c	CHL TDS	0.123	0.110 (ns)
H2a	COM CNT	0.193	0.001**
H2b	COM CON	0.322	<0.001***
H2c	COM TDS	0.312	<0.001***
H3a	NOV CNT	0.389	<0.001***
H3b	NOV CON	0.201	0.015*
H3c	NOV TDS	0.341	<0.001***
H4a	ENV CNT	0.032	0.483 (ns)
H4b	ENV CON	0.242	<0.001***
H4c	ENV TDS	0.124	0.051 (ns)
H5a	SOC CNT	0.241	<0.001***
H5b	SOC CON	0.145	0.043*
H5c	SOC TDS	0.091	0.241 (ns)
H6a	RLX CNT	−0.454	<0.001***
H6b	RLX CON	−0.244	<0.001***
H6c	RLX TDS	−0.392	<0.001***

*p < 0.05; **p < 0.01; ***p < 0.001; ns, not significant.

Challenge Motivation (CHL): Contrary to expectations, challenge motivation demonstrated no statistically significant impact on the three dimensions of flow experience. Specifically, its effects on Concentration (CNT) (β = 0.148, *p* = 0.064), Sense of Control (CON) (β = 0.050, *p* = 0.468), and Time Distortion (TDS) (β = 0.123, *p* = 0.110) were non-significant. Consequently, H1a, H1b, and H1c are not supported.

Competition Motivation (COM): As hypothesized, competition motivation exerted a robust and significant positive influence on all flow dimensions. The data support H2a (CNT: β = 0.193, *p* = 0.001), H2b (CON: β = 0.322, *p* = 0.000), and H2c (TDS: β = 0.312, *p* = 0.000).

Novelty and Exploration Motivation (NOV): This construct was found to be a strong positive predictor of flow experience. Significant effects were observed for CNT (β = 0.389, *p* = 0.000), CON (β = 0.201, *p* = 0.015), and TDS (β = 0.341, *p* = 0.000), confirming the validity of H3a, H3b, and H3c.

Natural Environmental Experience Motivation (ENV): The analysis revealed a selective impact where environment motivation significantly enhanced the participant’s Sense of Control (CON) (β = 0.242, *p* = 0.000), supporting H4b. However, its influence on CNT (β = 0.032, *p* = 0.483) and TDS (β = 0.124, *p* = 0.051) did not reach statistical significance.

Social Interaction Motivation (SOC): Social drivers positively influenced Concentration (CNT) (β = 0.241, *p* = 0.000) and Sense of Control (CON) (β = 0.145, *p* = 0.043), thus supporting H5a and H5b. The path to Time Distortion (TDS) was non-significant (β = 0.091, *p* = 0.241).

Relaxation Motivation (RLX): Interestingly, relaxation motivation exhibited a significant negative effect on all flow experience dimensions. Significant negative paths were recorded for CNT (β = −0.454, *p* = 0.000), CON (β = −0.244, *p* = 0.000), and TDS (β = −0.392, *p* = 0.000). This suggests that a primary focus on leisure may inhibit deep immersion, supporting H6a, H6b, and H6c as negative relationships.

#### 3.2.3 Summary of explanatory power

The integrated model elucidates the varying roles that diverse motivational drivers play in shaping the psychological state of sailors. The R^2^ values for the endogenous constructs were 0.552 for Concentration, 0.398 for Sense of Control, and 0.506 for Time Distortion. According to Cohen (2013), these R^2^ values indicate moderate to substantial explanatory power. These coefficients of determination indicate that the model possesses substantial explanatory power and is statistically significant (Cohen, 2013). While achievement-oriented and discovery-based motivations (COM and NOV) act as primary catalysts for flow, relaxation-seeking (RLX) appears to be inversely related to the intense concentration required for a flow state during sailing competitions.

## 4 Discussion

### 4.1 Discussion and practical implications

The empirical findings of this study provide important insights into the motivational antecedents of flow experience in competitive sailing contexts, highlighting the psychological complexity underlying maritime sport participation. The results demonstrate that different motivational orientations exert distinct influences on flow experience, supporting the context-dependent nature of flow proposed in sport psychology.

Among the motivational factors examined, competition motivation and novelty–exploration motivation emerged as the most robust predictors of flow experience. Competition motivation significantly influenced all three flow dimensions, particularly sense of control and time distortion. In sailing regattas, competitive environments provide clear performance goals and immediate feedback, which help structure attentional focus and facilitate an “ordered consciousness” conducive to flow. This finding is consistent with flow theory, which emphasizes the importance of goal clarity and feedback in sustaining deep immersion during performance activities. Similarly, novelty and exploration motivation was found to be the strongest predictor of concentration. Competitive sailing is characterized by environmental uncertainty and rapidly changing conditions, and sailors motivated by exploration are more likely to allocate sustained attentional resources to monitoring wind shifts, sea states, and tactical cues. Such heightened vigilance reinforces task absorption and cognitive engagement, thereby creating favorable conditions for flow experiences.

In contrast, relaxation motivation demonstrated a consistent and significant negative relationship with all flow dimensions, particularly concentration. This seemingly counterintuitive result can be explained by a psychological mismatch between relaxation-oriented motives and the arousal demands of competitive sport environments. While sailing is often associated with leisure and recreation, regatta participation requires sustained mental effort, heightened alertness, and continuous tactical decision-making. Relaxation motivation, which prioritizes tension reduction and mental disengagement, may therefore conflict with the level of cognitive engagement required to achieve flow. Participants driven primarily by relaxation may consciously or unconsciously avoid the intense attentional involvement necessary for deep immersion, leading to reduced concentration and perceived control (Akyol and Imamoglu, 2019; Harris et al., 2023; Sonnentag and Fritz, 2007).

Another noteworthy finding concerns challenge motivation, which did not significantly influence any dimension of flow experience. This result suggests that in competitive sailing contexts, perceived challenge alone may not be sufficient to induce flow unless it is accompanied by clear performance benchmarks or intrinsic engagement through exploration and learning. According to flow theory, optimal experience emerges when perceived challenge is balanced with perceived skill. When challenge levels exceed individuals’ perceived capabilities, anxiety may occur rather than immersion. In offshore sailing regattas, environmental uncertainty and performance risk are persistent rather than episodic, potentially normalizing the perception of challenge and diminishing its direct motivational salience. Similar patterns have been reported in previous sport psychology studies, where challenge was found to exert indirect or conditional effects on flow rather than a direct positive influence (Jackson et al., 2001; Peifer et al., 2014; Swann et al., 2012; Swann et al., 2015).

Taken together, these findings highlight the importance of distinguishing between motivational orientations that facilitate deep psychological engagement and those that may conflict with the cognitive and attentional demands of competitive sport participation. Achievement-oriented motivations and exploration-driven motives appear particularly conducive to flow experiences in competitive sailing environments, whereas relaxation-oriented motives may inhibit immersion. By clarifying these differential psychological mechanisms, this study contributes to sport psychology by extending our understanding of how motivation shapes flow experience in high-demand and uncertainty-driven competitive environments.

From a practical perspective, the findings suggest several strategies that may help foster stronger flow experiences in sailing competitions. Event organizers and sailing clubs may enhance competition motivation by designing race formats that emphasize clear performance goals, transparent feedback mechanisms, and opportunities for skill comparison among participants. Such elements can strengthen goal orientation and encourage deeper engagement during sailing competitions.

In addition, exploration and novelty motivations may be stimulated by incorporating varied race courses, changing sailing environments, or tactical challenges that encourage participants to actively monitor environmental conditions and experiment with different sailing strategies. These elements may enhance curiosity and attentional engagement, which are conducive to flow experiences.

At the same time, the results suggest that relaxation-oriented motivations may not align well with the attentional demands of competitive sailing. To mitigate this effect, sailing events may differentiate between recreational sailing activities and competitive regattas, allowing participants seeking relaxation to engage in non-competitive sailing experiences while maintaining high levels of challenge and focus in competitive events.

Finally, demographic characteristics may also shape motivational orientations and flow experiences. For instance, younger or less experienced sailors may be more motivated by exploration and skill development, whereas more experienced participants may place greater emphasis on competition and performance outcomes. Gender differences may also influence participation motivations in sport contexts. Future research could further investigate how demographic characteristics interact with motivational orientations to influence flow experiences in sailing participation.

### 4.2 Limitations

Several limitations of this study should be acknowledged when interpreting the findings. First, the sample was predominantly male, reflecting the current demographic structure of competitive sailing participation in Taiwan. However, this gender imbalance may limit the generalizability of the findings to more gender-balanced sport contexts. In addition, purposive sampling was used to recruit participants with sailing competition experience. While this approach ensured that respondents possessed relevant experience for the research context, it may also limit the extent to which the findings can be generalized to the broader population of recreational sailors.

Second, demographic characteristics such as age, sailing experience, and competition level may influence motivational orientations and flow experiences in sport participation. These variables were not included as control variables in the present analysis, as the study primarily focused on examining the motivational determinants of flow experience. Future research could incorporate these factors as control variables or moderators to better understand how motivational pathways to flow vary across participant groups and levels of sailing experience.

Third, although procedural and statistical steps were taken to minimize the potential influence of common method bias, the study relied on a single-source self-report survey design. As a result, some degree of method-related bias may still exist, and the findings should therefore be interpreted with appropriate caution.

Finally, a limitation relates to the cultural adaptation of the measurement scales. Although the questionnaire items were translated and back-translated to ensure linguistic equivalence, a full cultural adaptation process was not conducted. Because the scales were originally developed in different cultural contexts, certain motivational constructs may manifest differently across cultures. Future research could conduct cross-cultural validation or develop culturally grounded measurement instruments to better capture the contextual characteristics of sailing participation in Taiwan.

## 5 Conclusion

This study advances sport and performance psychology by clarifying how different participation motivations shape flow experience in a demanding competitive sport context. The findings demonstrate that motivational orientations do not exert uniform effects on flow; instead, competition-oriented and novelty-driven motivations play a central role in facilitating deep psychological immersion, whereas relaxation-oriented motivation appears incompatible with the attentional and control demands of competition. Notably, challenge motivation alone does not directly predict flow, suggesting that in high-demand sport environments, challenge may be normalized or perceived as pressure rather than as a catalyst for optimal experience.

By empirically distinguishing the differential motivational pathways to flow, this study extends flow theory by emphasizing its contextual sensitivity in competitive settings characterized by uncertainty and sustained performance demands. The results underscore the importance of aligning motivational orientations with situational requirements to foster optimal psychological engagement. Overall, the study contributes to a more nuanced understanding of motivation–flow dynamics in sport, offering theoretical insights relevant to athletes, coaches, and researchers interested in psychological engagement and performance under competitive conditions. Therefore, the findings should be interpreted within the context of the study’s methodological design and sample characteristics, and caution should be exercised when generalizing the results beyond the specific research context to broader populations.

## Data Availability

The raw data supporting the conclusions of this article will be made available by the corresponding author upon reasonable request.
